# Photobonding of silk fibroin-based hydrogels to rabbit corneas

**DOI:** 10.3389/fbioe.2025.1739461

**Published:** 2026-01-06

**Authors:** Rocio Gutierrez-Contreras, Patricia Gallego-Muñoz, Andrés De La Hoz, Mar Fernández-Gutierrez, Irene E. Kochevar, M. Carmen Martínez-García, Susana Marcos

**Affiliations:** 1 Instituto de Óptica “Daza de Valdés”, Consejo Superior de Investigaciones Científicas (IO-CSIC), Madrid, Spain; 2 Departamento de Biología Celular, Genética, Histología y Farmacología, GIR de Técnicas Ópticas de Diagnóstico, Universidad de Valladolid, Valladolid, Spain; 3 Wellman Center for Photomedicine, Massachusetts General Hospital, Harvard Medical School, Boston, MA, United States; 4 Center for Visual Science, The Institute of Optics, Flaum Eye Institute, University of Rochester, Rochester, NY, United States

**Keywords:** cornea, hydrogels, photobonding, re-epithelialization, silk fibroin, sutureless

## Abstract

**Introduction:**

Corneal abrasions are common ocular injuries characterized by the loss of epithelial cells. Severe cases are often treated with amniotic membrane transplantation. However, as an allogenic tissue, it can trigger immune responses, it is scarce and costly, and may require suturing to the cornea. In this study, we propose and evaluate two silk fibroin-based hydrogels implanted in rabbit corneas with a sutureless photobonding technique as a surrogate for the amniotic membrane in corneal wound healing.

**Methods:**

Silk fibroin-based hydrogels were developed with polyethylene glycol (PEG) 300 or 3350. The hydrogels were stained with 0.01% Rose Bengal and photobonded to *ex vivo* de-epithelialized rabbit corneal strips using a custom-developed irradiation system (532 nm; 0.13 or 0.15 W/cm^2^ irradiance for 6.6 min). Bonding strength after 24 and 72 h under hydrated conditions was measured using a uniaxial stretcher, with five samples per experiment. An *in vivo* proof of concept study was also performed: hydrogels were implanted in four anesthetized rabbits that were euthanized immediately after photobonding for bonding assessment after 24 and 72 h in hydration. Two additional rabbits received *in vivo* implants, were clinically monitored for 15 and 30 days, and euthanized for histological evaluation.

**Results:**

Bonding strengths measured 24- or 72-h after irradiation at 0.13 or 0.15 W/cm^2^ of 532 nm light reached 2–3 N/cm^2^ for both types of Rose Bengal-stained hydrogels. *In vivo* bonding strength was consistent with the *ex vivo* results. At 15 and 30 days after the procedure, the corneas exhibited complete re-epithelialization beneath the hydrogel.

**Conclusion:**

Silk fibroin-based hydrogels can be successfully bonded to *ex vivo* rabbit corneas using a sutureless photobonding technique, achieving high bonding strength. The *in vivo* proof-of-concept study demonstrated the feasibility of the surgical procedure and confirmed corneal re-epithelialization.

## 1 Introduction

Amniotic membrane (AM) is extensively used in ophthalmology for corneal regeneration in persistent epithelial defects, functioning as a patch, as a graft or a combination of both. It facilitates re-epithelialization and exhibits anti-fibrotic, anti-inflammatory, anti-angiogenic, and anti-microbial properties (Jirsova and Jones, 2017; Walkden, 2020).

Typically, the AM is sutured to the cornea; however, sutureless approaches such as Prokera or fibrin glue are also employed in clinical practice. The sutureless techniques offer advantages including easier surgical manipulation during the surgery, reduced suture-related complications, and shorter surgical time (Kucukerdonmez et al., 2010; Walkden, 2020; Baykara et al., 2022). In this regard, it has been demonstrated that the AM can be bonded to the rabbit cornea through a sutureless light-initiated method (Verter et al., 2011; Mohammad et al., 2024).

Despite its advantages, including the presence of growth factors and anti-inflammatory and anti-angiogenic properties, the AM’s scarcity, procurement challenges, and allogenic nature contribute to its high cost and the need to seek for alternatives (Ma et al., 2010). Consequently, silk fibroin (SF)-based biomaterials have garnered interest for biomedical applications (Murphy and Kaplan, 2009; Lammel et al., 2010; Zhou et al., 2017; Ciocci et al., 2018; Nguyen et al., 2019), and the current study explores its potential to replace AM in corneal wound healing treatments. SF, derived from silkworm silk, is abundant, easily obtainable, and biocompatible (Vepari and Kaplan, 2007). It enhances corneal healing (Tran et al., 2019) and can be made into transparent hydrogels. SF is composed of fibroin (70–75 wt%) and sericin (30%–25%) (Huang et al., 2023; Välisalmi and Linder, 2024).

For biomaterial implants, biodegradability or bioresorption, alongside biocompatibility, is crucial depending on the intended application. SF, an FDA-approved biomaterial, is classified as non-biodegradable material by the US Pharmacopeia, though it can be enzymatically degraded. The proteolytic degradation of SF can be modulated by adjusting processing parameters and crystallinity (Gutierrez-Contreras et al., 2024). Degradation time varies with material content, secondary structure, processing conditions and implantation site characteristics (Cao and Wang, 2009; Kundu et al., 2013; Guo et al., 2020).

SF’s properties and versatility allow for development of various formats, including films, sponges, gels and nanoparticles (Rockwood et al., 2011), that are tailored to specific applications. A SF hydrogel as a cost-effective accessible alternative to AM is proposed in this study, offering comparable biological properties (Suzuki et al., 2019). Previous research has demonstrated the potential of silk proteins for ocular tissue reconstruction (Manoochehrabadi et al., 2025), for instance, with human corneal limbal epithelial cells grown on methanol cross-linked hydrogels (Chirila et al., 2007; Chirila et al., 2008).

In this study, SF hydrogels were formed through polyethylene glycol (PEG)-induced gelation. PEG is a reported porogen (Suzuki et al., 2019). However, previous work has shown that, due to its high affinity for water, PEG also induces local dehydration of SF, promoting the transition from random coil to beta-sheet structures, and thereby increasing crystallinity (Um et al., 2003; Wang et al., 2015; Gutierrez-Contreras et al., 2024). Here, we have evaluated SF hydrogels as potential corneal dressings. As an alternative fixation strategy, we used a sutureless, light initiated method based on the photosensitizer Rose Bengal (RB) and green light (De la Hoz A, et al. IOVS 2019; 60:ARVO E-Abstract 3218; Gutierrez-Contreras R, et al. IOVS 2023; 64:ARVO E-Abstract 3126) (Verter et al., 2011; Gutiérrez Contreras et al., 2022). This technique had been applied previously *in vivo* in a rabbit model to stiffen the corneal stroma collagen, with the ultimate goal of halting keratoconus progression in patients. The post- RB/green light irradiation study proved effective and safe in the rabbit model (Zhu et al., 2016; Gallego-Muñoz et al., 2017; Lorenzo-Martín et al., 2018).

In this study, we demonstrate the feasibility of bonding silk-fibroin hydrogel membranes to rabbit corneal tissue via a light-initiated method, and provide proof-of-concept of *in vivo* photobonding of these membranes in a rabbit model with successful wound healing.

## 2 Methods

### 2.1 Animals, materials and reagents

Freshly enucleated (within 24 h) young rabbit eyes (from 2-month-old commercial hybrid rabbits of undisclosed sex) provided by Grupo Hermi slaughterhouse (Valladolid, Spain) were used for *ex vivo* experiments. Six four-month-old female *New Zealand* albino rabbits (3–4 kg weight) were obtained from Granja San Bernardo (Navarra, Spain), an approved and officially registered supplier of laboratory animals. The animals were used for the *in vivo* evaluation of SF hydrogel bonding to the cornea. The study protocols on *in vivo* rabbits were approved by the Animal Research and Welfare Ethics Committee of the University of Valladolid-Spain (Reference Number: 12905683) in agreement with European (Council Directive, 2010/63/UE) and Spanish regulations (RD 53/2013). Animals were handled following the guidelines of Animal Research: Reporting of *In Vivo* Experiments (ARRIVE) and the guidelines of the Association for Research in Vision and Ophthalmology (ARVO) Statement for the Use of Animals in Ophthalmic and Vision Research.

All SF hydrogels were prepared from *Bombyx mori* silk cocoons, harvested at Instituto Murciano de Investigación Agraria y Medioambiental (IMIDA) (Murcia, Spain).

The following reagents were used to extract the SF from the silk cocoons and prepare the hydrogels: sodium carbonate anhydrous 99.5% with a laboratory reagent grade, supplied by Thermo Fisher Scientific, USA. PEG 300 for synthesis, powder PEG 3350, PBS in tablets, lithium bromide ReagentPlus(R) >99% and Rose Bengal (RB, 95%) were supplied by Merck, Germany. Dialysis membrane MWCO 3.5 kDa was supplied by Spectrum Chemical, United States.

The reagents used to fix the corneas were buffered paraformaldehyde, supplied by Acros Organics, Germany; paraffin, supplied by Labkem, Spain; and haematoxylin-eosin, supplied by Fisher Bioreagent, Belgium.

### 2.2 Preparation of SF hydrogels

SF was prepared following the general procedure previously published (Rockwood et al., 2011), with some modifications as explained in a recent work carried out in our group (Gutierrez-Contreras et al., 2024). In brief, SF was degummed for 40 min and washed three times in distilled water. The degummed SF fibers were dried at 60 °C overnight. SF was then dissolved in 9.3M LiBr solution for 4 h at 60 °C. Throughout this time, the mix was stirred twice with a spatula to ensure complete dissolution of SF in the LiBr solution. The SF-LiBr solution was dialyzed in a 16 cm membrane, folded three times on each side before clamping. Then, the dialyzed SF solution was centrifuged at 7830 RPM for 1 h at 4 °C to remove impurities.

Two different types of hydrogels were prepared with SF solution and PEG 300 or PEG 3350, respectively. 3 mL of a 3% SF (w/v) solution was stirred either with 5% PEG 300 (v/v) (SF-PEG300 hydrogel) or 450 µL of 30% PEG 3350 (w/v) (SF-PEG3350 hydrogel) at 700 RPM for 2 min. Then, the solutions were cast onto 90-mm diameter lidless polystyrene petri dishes. For the SF-PEG300 hydrogel, 0.3 mL of water were added to the SF-PEG 300 solution and stirred before casting. The casting was carried out overnight in a climatic chamber (Memmert HPP 260 eco) at 25 °C and 40% relative humidity, with the petri dish placed on an orbital shaker at 40 RPM. After casting, the hydrogels were washed in water overnight, to remove the PEG. SF hydrogels have been previously characterized in a recent publication by our group (Gutierrez-Contreras et al., 2024). SF-PEG300 and SF-PEG3350 hydrogels were cut into 8 × 10 mm strips. The corners of the hydrogel strips were beveled to enhance their conformity to the corneal surface. Hydrogels strips were stained for 10 min in a 0.01% (w/v) RB solution in PBS.

### 2.3 Photobonding of SF hydrogel strips to rabbit corneas

#### 2.3.1 *Ex vivo* photobonding

A custom-developed illumination system was used for photobonding. The light source is a diode pumped solid-state green (532 nm) laser (Frankfurt Laser, Friedrichsdorf, Germany), coupled to a fiberoptic and a 75-mm focal length lens that collimates the light, producing a 12-mm disk at the corneal plane.

**FIGURE 1:**
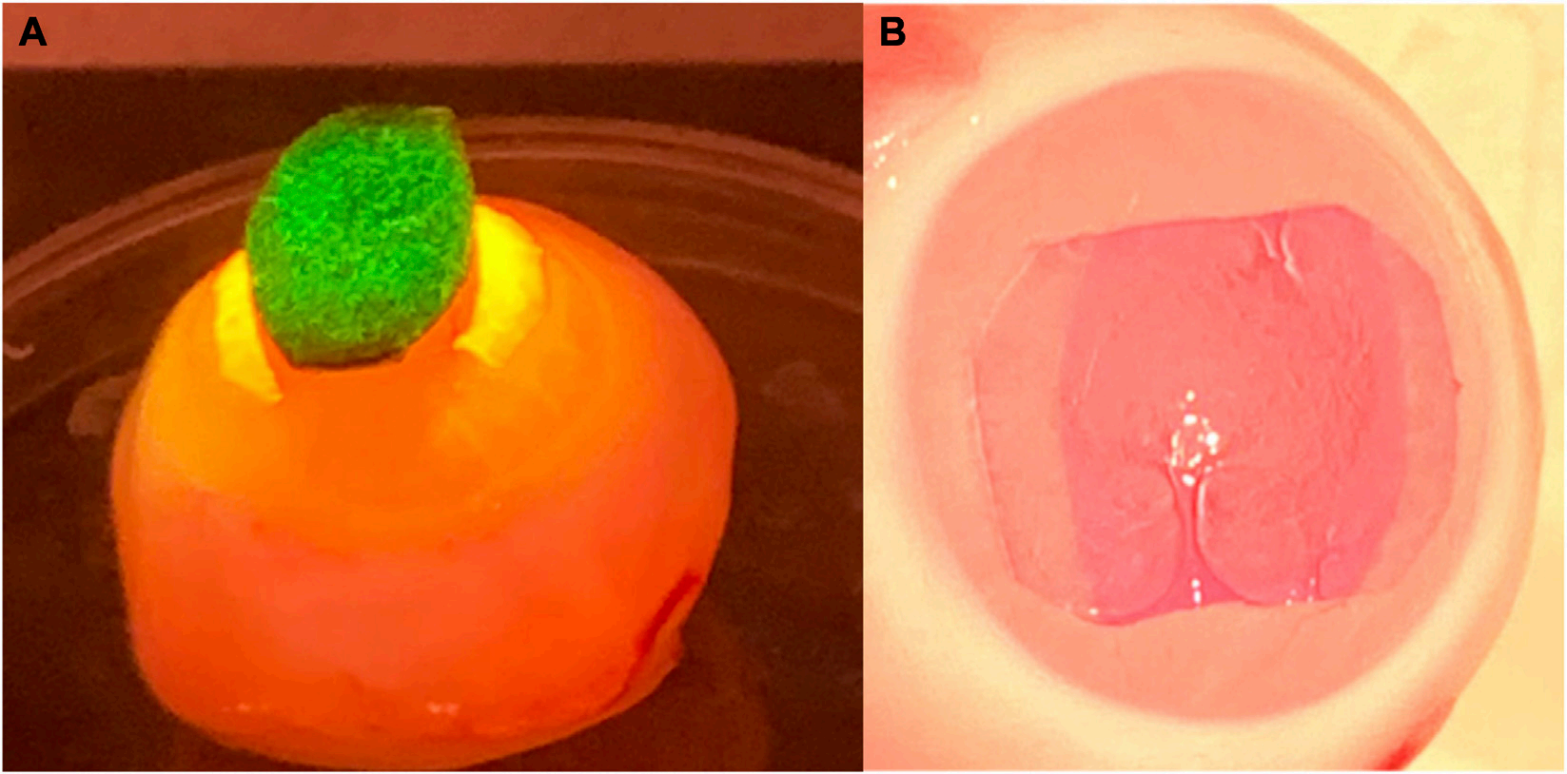
SF hydrogel-cornea photobonding in *ex vivo* eyes. **(A)** Oblique lateral view of the eye during irradiation with a shielding pupil mask (showing the reflected green irradiation light). The edges of the RB-stained SF hydrogel absorb green light, showing a yellowish color during irradiation. **(B)** Frontal view of the eye after irradiation, the RB-stained SF hydrogel showing photobleached edges.

Rabbit eyes (n = 5 per experiment, 80 eyes in the entire experiment) were used 24 h after enucleation and the tissue around the eyeball was removed. The cornea was completely de-epithelialized by scraping it with a scalpel immediately before the hydrogel photobonding treatment. The rabbit eye was placed with the cornea facing up. The hydrogel strip was taken out of the RB solution and excess RB was removed from the strip with a paper towel, and placed centrally over the cornea, perpendicularly to the nasal - temporal axis. An oval (10-mm length and 6-mm central width) pupil mask was placed on top of the strip, perpendicular to it. The pupil mask shields light from reaching the retina and also allows to irradiate only the ends of the hydrogel strip (Figure 1A). Additionally, an opaque frame in the shape of the strip was placed between the laser and the sample, after the collimating lens, to shield light from reaching the limbus and allow for a good centration of the cornea with the laser spot. Two light irradiances were tested: 0.13 and 0.15 W/cm^2^. All samples were irradiated for 6.6 min yielding fluences of 51.5 and 59.4 J/cm^2^, respectively. These fluences have been shown in our previous study to bond amniotic membrane to de-epithelialized rabbit cornea (Verter et al., 2011). Two different types of hydrogels were used (SF-PEG300 and SF-PEG3350 strips) and photobonded to the rabbit eyes (n = 5 per hydrogel type). Photobleaching occurred at the edges of the hydrogel (1–1.5 mm wide), consistent with reported observations of RB photochemistry and photo-bonding/cross-linking reactions mediated by RB (Alarcon et al., 2017) (Figure 1B). Subsequently, the eye was positioned with the cornea facing downward in a container lined with a paper towel soaked in PBS and covered with the lid to maintain humidity conditions comparable to those *in vivo*.

The eye was left at 4 °C for 24 or 72 h. These time points were chosen to represent short- and long-term intervals, following photobonding. The 24-h mark was established as the lower limit, as the hydrogel’s expected residence time on the *in vivo* cornea exceeds this period. Conversely, 72 h was selected as the upper timepoint, since longer incubation would result in tissue degradation in the fresh *ex vivo* model.

#### 2.3.2 *In vivo* photobonding

To translate the surgical technique to an *in vivo* setting, a proof-of-concept experiment was conducted in rabbits *in vivo.* Two groups of animals were used. The first group consisted of four rabbits, each receiving photobonding in both eyes with the same hydrogel—two with SF-PEG300 and two with SF-PEG3350. These animals were euthanized following irradiation to evaluate the bonding strength after *in vivo* photobonding.

After enucleation, the eyes were kept in PBS at 4 °C for 24–72 h prior to bonding strengths measurements. The second group included two rabbits, and a SF-PEG300 hydrogel strip was photobonded to one eye of both rabbits; the contralateral eyes were used as controls. These animals were clinically followed and euthanized at 15- and 30-day post-treatment, respectively, to assess the re-epithelialization under the hydrogel by histological evaluation.

**FIGURE 2:**
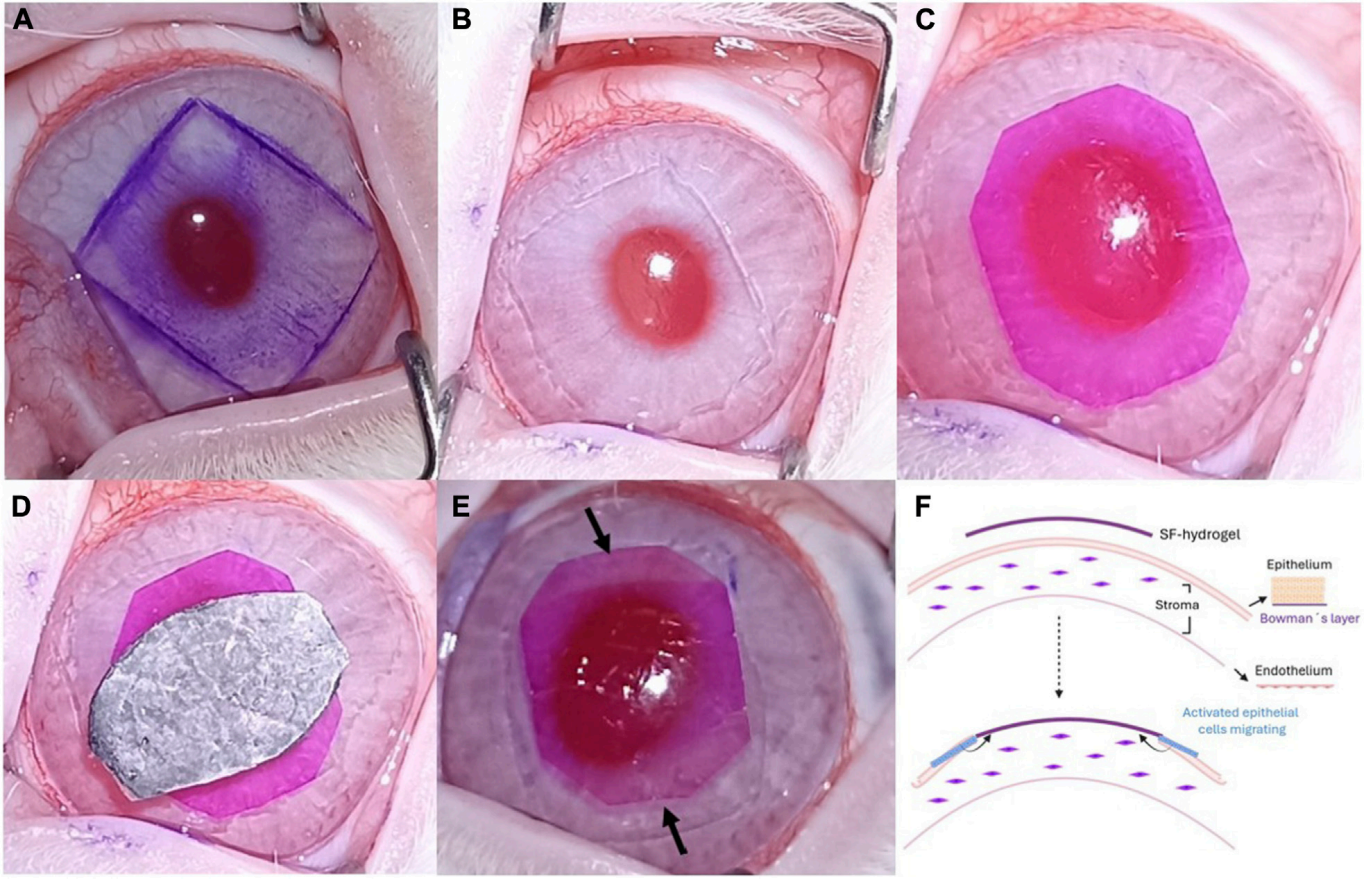
SF hydrogel-cornea photobonding in *in vivo* eyes. **(A)** Marked zone for epithelium removal, stained with skin pen ink. **(B)** De-epithelialized cornea. **(C)** RB-stained SF strip hydrogel on top of the de-epithelialized area. **(D)** Optical pupillary zone shielded from irradiation. **(E)** Photobonded SF strip hydrogel to cornea. The arrows show photobleaching in the top and bottom hydrogel edges, following irradiation. **(F)** Schematic representation of the expected effect of the hydrogel on the corneal cells surface, including cells underneath the hydrogel.

The procedure was performed under general anesthesia with a thigh intramuscular injection of ketamine hydrochloride (7.5 mg/kg; Anesketin 100 mg/mL, Dechra, Netherlands) and medetomidine hydrochloride (0.225 mg/kg; Sedator 1 mg/mL, Dechra, Netherlands), in a 1:3 ratio, at a total dose of 300 μL/kg, followed by topical application of 0.5% tetracaine hydrochloride and 1 mg of oxybuprocaine (Colircusi Anestésico Doble, Alconcusí SA, Barcelona, Spain). The first step of the procedure involved partial corneal de-epithelialization. A surgical skin marker and a rubber stamp were used to create a pupil-centered 8 × 10 mm central mark on the area designated for de-epithelialization (Figure 2A), aligned perpendicularly to the nasal-temporal axis. The corneal epithelium within the marked region was then carefully removed by gentle scraping with a sharp blade, allowing the SF hydrogel strip to be placed directly onto the exposed stromal collagen, where cross-linking occurs (Figure 2B). The RB-stained SF strip was placed over the de-epithelialized corneal area (Figure 2C). To prevent neovascularization, the strip was positioned 2–3 mm away from the limbus. Irradiation was performed following the same protocol as the *ex vivo* photobonding, using an irradiance of 0.15 W/cm^2^ for 6.6 min, corresponding to a fluence of 59.4 J/cm^2^ (Figure 2D). This treatment resulted in visible bleaching of the RB dye after irradiation (Figure 2E). The animals were euthanized under general anesthesia (as previously described above) by intracardiac injection of sodium pentobarbital (Dolethal 0737-ESP Vetoquinol, Madrid, Spain; Dose: 200 mg/kg).

### 2.4 Bonding strength measurements

**FIGURE 3:**
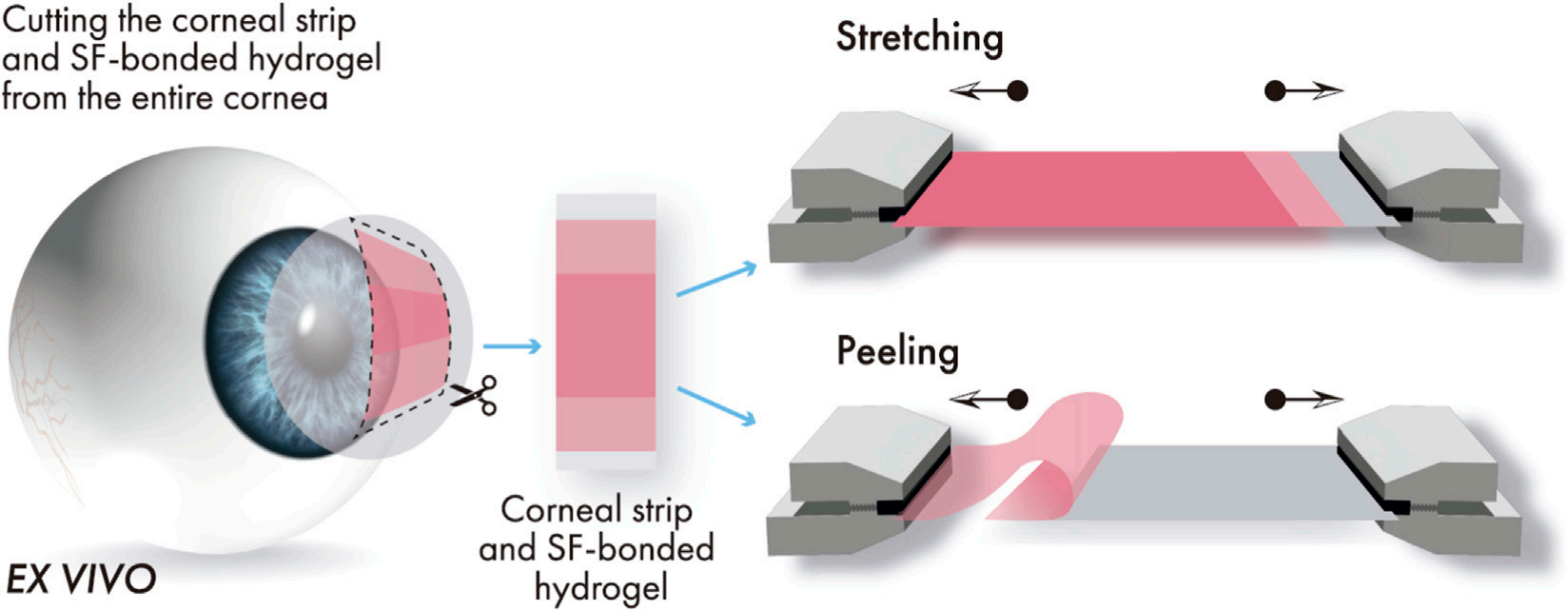
Cutting and mounting of the corneal-SF hydrogel strip in the uniaxial stretcher for measurement of stretching and peeling forces per unit area.

After 24 and 72 h in PBS hydration, the eyes (from both the *ex vivo* and *in vivo* photobonding procedures) were processed by cutting the corneal strip and SF-bonded hydrogel. The photobonded strip of cornea superimposed with the strip of the bonded SF-hydrogel was cut from the entire cornea, leaving a rim outside the bonded area (in the longer side of the sample) to allow mounting the sample on the stretcher device (Figure 3). Both ends of the hydrogel were photobonded to the cornea, but only one was selected for stretching or peeling tests to assess bonding forces per unit area (bonding strength).

Stretching strengths were taken from the maximum force recorded in the uniaxial stretcher (UStretch, Cellscale, Waterloo, ON, Canada) before detachment or breakage of the hydrogel from the corneal strip, divided by the bonding area. Peeling strength was quantified using the same set-up as the stretching test, but with a different sample orientation on the uniaxial stretcher.

In the stretching test, the unbonded ends of the cornea and the SF hydrogel were each clamped to opposing shoes and pulled apart. In the peeling test, the unbonded portion of the SF-hydrogel strip was folded along the corneal strip, clamped to the stretcher shoe, and peeled off (Figure 3). Careful alignment of the samples on the uniaxial stretcher was essential to avoid premature peeling during mounting. All stretching and peeling tests were conducted under distilled water immersion to prevent artifacts associated to dehydration during irradiation. Each test was completed within 1 min per sample; therefore, swelling during the measurement was considered negligible.

### 2.5 Clinical follow-up and histology

The clinical assessment of the SF-hydrogel implantation was performed on the anterior segment of the eye under a surgical microscope (Leica M220 F12; Leica Microsystems, Nussloch, Germany) following the described surgical procedure. Monitored clinical parameters included neovascularization development, loss of transparency, hydrogel stability, and adhesion.

Both rabbits were observed daily during the first post-operative week and again on day 15 following SF hydrogel bonding. One rabbit was euthanized on day 15, while the other was monitored until day 30.

Immediately after euthanasia, corneas were removed for histological follow up. The corneas were fixed in 4% buffered paraformaldehyde and embedded in paraffin. Corneal sections (5-µm thick) were deparaffined and stained with Haematoxylin-Eosin (H&E). The sections were examined under a Zeiss Axiophot HBO-50 (Carl Zeiss, Oberkochen, Germany) and photomicrographs were taken using a Leica DMC 6200 digital camera (Leica Microsystems AG Max Schmidheiny Strasse 201; 9435 Heerbrugg, Switzerland).

## 3 Results

### 3.1 Stretching and peeling strengths

**FIGURE 4:**
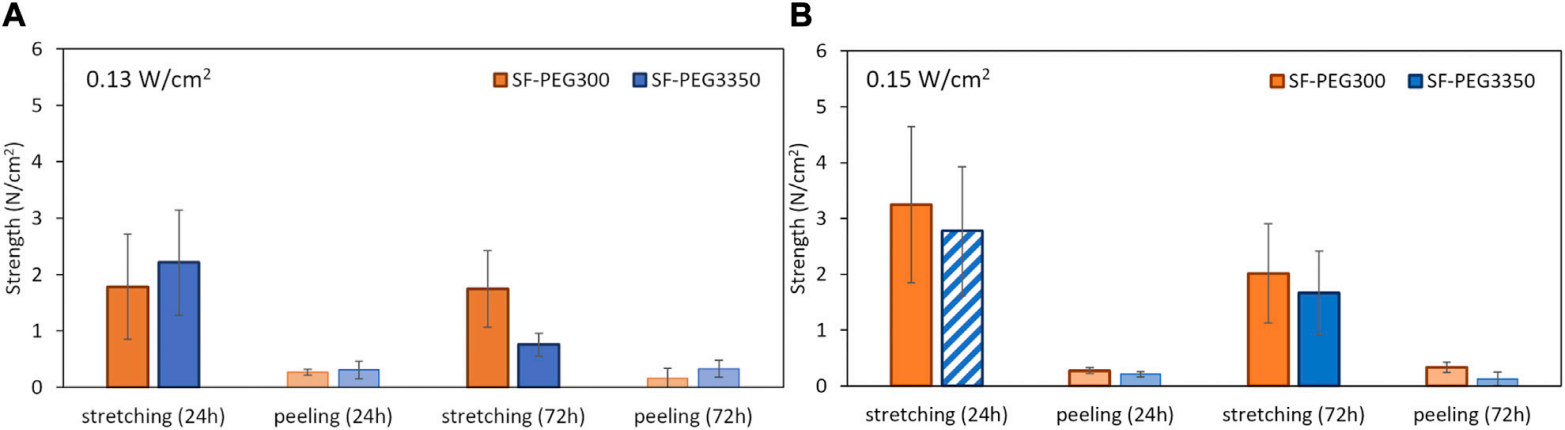
Bonding strengths of SF-PEG300 (orange bars) and SF-PEG3350 (blue bars) hydrogels to cornea at 24 and 72 h post-photobonding. Dark bars represent stretching and light bars represent peeling. **(A)** Stretching and peeling strengths after 0.13 W/cm^2^ irradiation. **(B)** Stretching and peeling strengths after 0.15 W/cm^2^ irradiation. The striped bars indicate hydrogels that fractured before detachment from the cornea; in these cases, the reported value corresponds to the hydrogel’s breakage strength.

**FIGURE 5:**
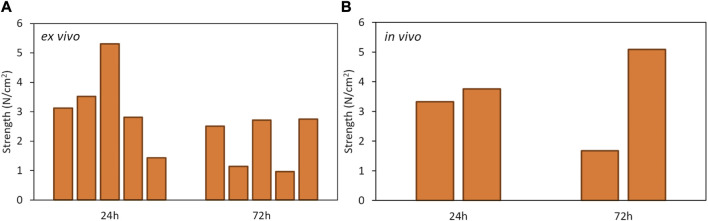
Stretching strengths of SF-PEG300 hydrogels 24 and 72 h post-photobonding. **(A)** Photobonding performed *ex vivo* at 0.15 W/cm^2^ irradiation. **(B)** Photobonding performed *in vivo* at 0.15 W/cm^2^ irradiation.

**FIGURE 6:**
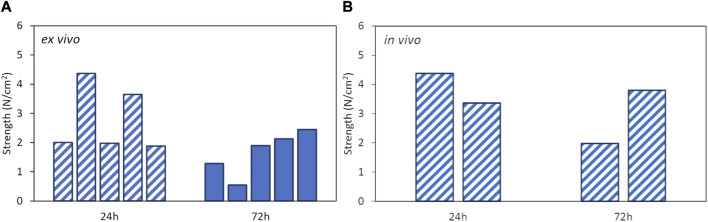
Stretching strengths of SF-PEG3350 hydrogels 24 and 72 h post-photobonding. **(A)** Photobonding performed *ex vivo* at 0.15 W/cm^2^ irradiation. **(B)** Photobonding performed *in vivo* at 0.15 W/cm^2^ irradiation. The striped bars indicate hydrogels that broke before detaching from the cornea.

Bonding strengths measured in the uniaxial stretcher are presented in Figures 4–6, in samples from *ex vivo* photobonding (Figures 4, 5A, 6A) and *in vivo* photobonding (Figures 5B, 6B) experiments.

A four-way ANOVA revealed that the material type (SF-PEG300 and SF-PEG3350) did not show a significant effect on the measured response. Consequently, a three-way ANOVA excluding the material factor was carried out, which showed that the assay type (stretching vs*.* peeling) was the main factor influencing the bonding strength (*R* ^2^ = 0.7; statistical power = 1.00). Levene’s test confirmed homogeneity of variances, allowing for the use of Student’s *t*-tests to further explore pairwise group differences. The t-test analysis confirmed that the stretching strengths (0.76–3.24 N/cm^2^) were significantly larger (p < 0.05) than peeling strengths (0.13–0.33 N/cm^2^) in all conditions: 6 times larger for 0.13 W/cm^2^ and 10 times larger for 0.15 W/cm^2^, on average across hydrogels and post-photobonding time (Figure 4). No statistical differences were seen between both types of irradiances. A decreasing trend in *ex vivo* stretching strength was observed from 24 to 72 h post-photobonding (Figure 4). This reduction reached statistical significance only for samples bonded under an irradiance of 0.15 W/cm^2^. In control corneas (non-irradiated), the stretching and peeling strengths were zero.

The stretching strengths found in the *ex vivo* and the *in vivo* photobonding tests were consistent: 3.2 ± 1.4 N/cm^2^ (24 h) and 2.0 ± 0.9 N/cm^2^ (72 h) in *ex vivo* experiments, and 3.5 ± 0.3 N/cm^2^ (24 h) and 3.4 ± 2.4 N/cm^2^ (72 h) in *in vivo* experiments, for SF-PEG300 at 0.15 W/cm^2^ (Figure 5); 2.8 ± 1.2 N/cm^2^ (24 h) and 1.7 ± 0.8 N/cm^2^ (72 h) in *ex vivo* experiments, and 3.9 ± 0.7 N/cm^2^ (24 h) and 2.9 ± 1.3 N/cm^2^ (72 h) in *in vivo* experiments, for SF-PEG3350 at 0.15 W/cm^2^ (Figure 6). *In vivo*, hydrogels fractured upon stretching before detachment from the cornea, indicating higher effective bonding strengths than those indicated by the reported values.

### 3.2 Long-term findings post-photobonding

**FIGURE 7:**
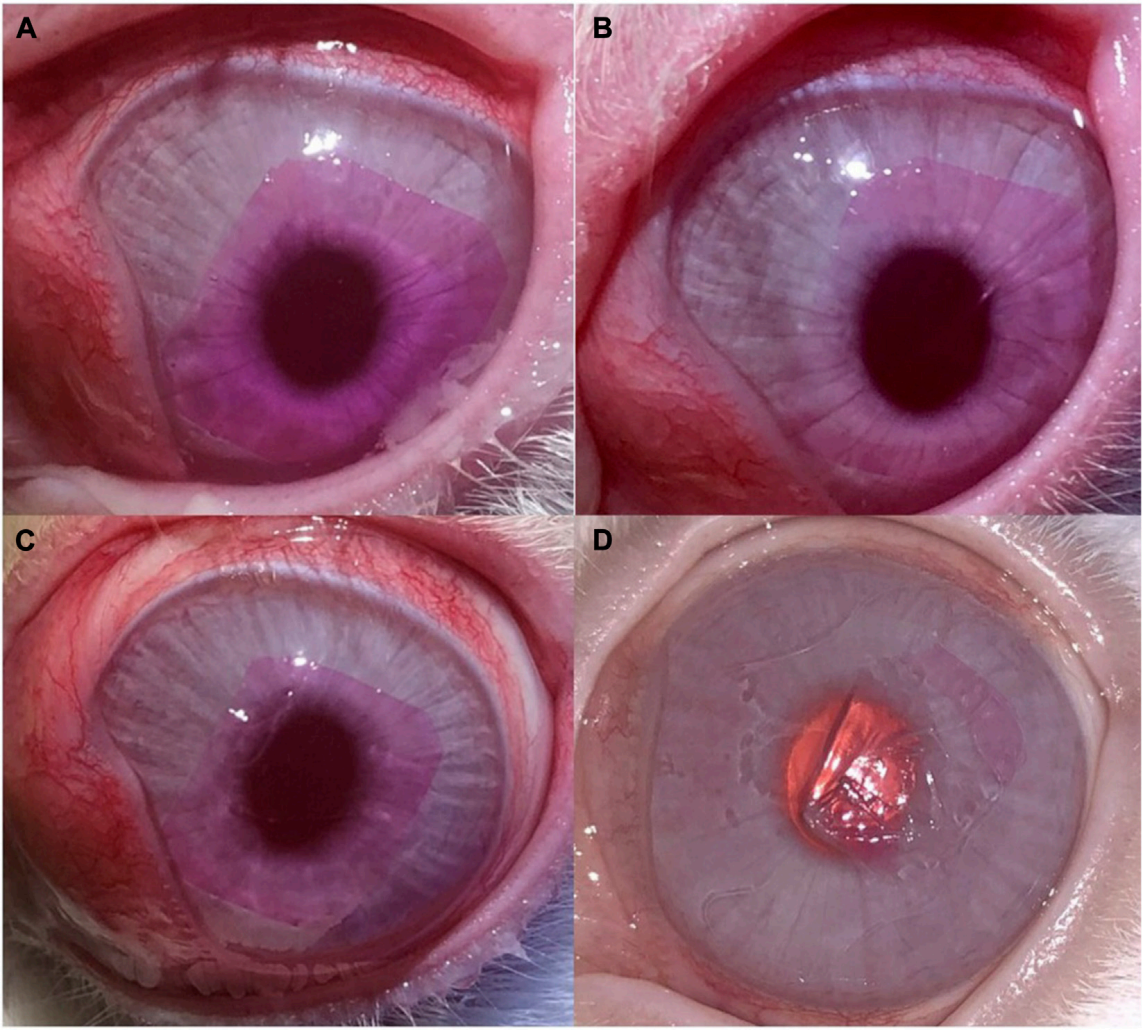
SF-PEG300 hydrogel bonded to the rabbit cornea. **(A)** 24 h post-photobonding. **(B)** 6 days post-photobonding. **(C)** 15 days post-photobonding. **(D)** 30 days post-photobonding.

Clinical follow-up showed that the SF-hydrogel (SF-PEG300) can remain bonded to the cornea surface 30 days after photobonding (Figures 7A–D), with the hydrogel remaining practically intact until day 15 (Figure 7C). At day 30, the hydrogel started to show some signs of fragmentation (Figure 7D). The hydrogel remained optically transparent despite being tinted with RB. The pink coloration of the hydrogel gradually diminished over time (from days 1, 6, 15 and 30 Figures 7A–D) as the dye was washed out by the tear film, leaving the material transparent and nearly colorless throughout time by the end of the observation period. No signs of discomfort—such as corneal scratching in the eye, mucus secretion or tearing—were observed, and the hydrogel was well tolerated throughout the study period.

**FIGURE 8:**
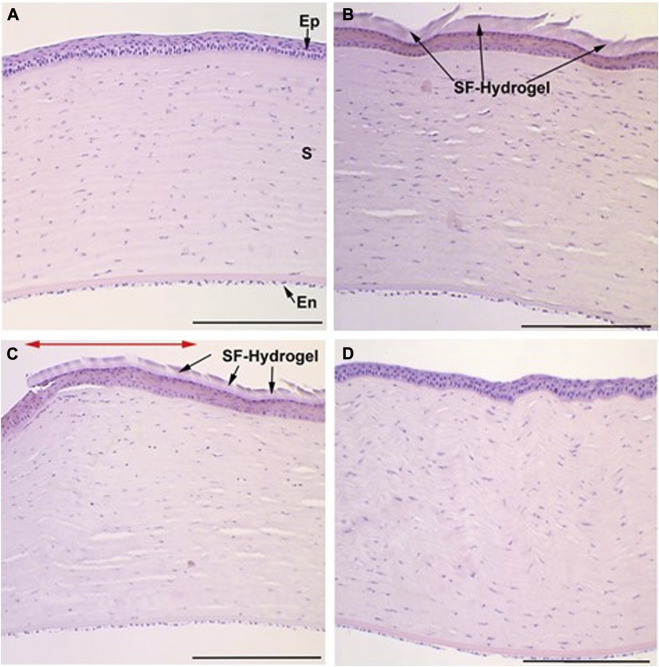
Corneal section stained with H&E, at different study time points after photobonding. **(A)** Control cornea. Labels stand for epithelium (Ep), stroma (S) and endothelium (En). **(B)** Center of the cornea with SF- hydrogel on the surface 15 days after photobonding (discontinuities observed in the hydrogel were produced when cutting the sample in preparation for the histological study). **(C)** Peripheral cornea around the photobonded area 15 days after photobonding. The double red arrow marks the zone of photobonding. The right side of the micrograph shows the SF-hydrogel and the epithelium regrowth underneath the hydrogel. **(D)** Central part of the cornea 30 days after photobonding. The SF-hydrogel was lost during histological processing of the sample; however, the regenerated epithelium exhibited morphology comparable to that of the observed control cornea. Scale bars: 200 µm.

The histological assays (Figure 8), 15 and 30 days after SF-hydrogel bonding show that complete re-epithelization occurred underneath the hydrogel, indicating proper cell migration and proliferation in the presence of the hydrogel (Figures 8B–D). As shown in Figure 8C, epithelial cells migrated beneath the membrane toward the central region (Figure 8B), consistent with the scheme in Figure 2F. One month after hydrogel adhesion (Figure 8D), the corneal histological structure closely resembled that of the control cornea (Figure 8A). No stromal alterations were observed at any of the time points examined following SF-hydrogel bonding.

## 4 Discussion

SF-based hydrogels were formed through PEG 300 or PEG 3350 induced gelation. These hydrogels exhibit critical characteristics desirable in ocular biomaterials: they are thin, transparent, flexible, yet sufficiently robust for surgical manipulation and to facilitate cell adhesion and migration. Following photobonding of the SF hydrogels to *ex vivo* corneas, the measured peeling strengths were consistent with those reported for photobonding of AM to de-epithelialized rabbit corneas (Verter et al., 2011), a benchmark for the current study. Importantly, the maximum fluence used in the current study (59.4 J/cm^2^) was approximately 2.5 times lower than the fluence used to seal AM to cornea in (Verter et al., 2011) and well below the established safety threshold (Zhu et al., 2016). A higher fluence was selected for the *in vivo* experiments than in *ex vivo* experiments to compensate for additional mechanical stresses acting on the photobonded hydrogel *in vivo* such as eyelid movement, the nictitating membrane and blinking, which are absent in *ex vivo* conditions.

Photobonding of AM to cornea is generally attributed to the formation of covalent bonds between collagen fibers in the two tissues, and a similar mechanism can be proposed for bonding of SF to cornea. RB acts as a photosensitizer that, upon green-light irradiation, reaches an excited triplet state that reacts with oxygen to generate singlet oxygen, a reactive oxygen species. Singlet oxygen can react with amino acids in collagen and SF, forming radicals that promote intermolecular cross-linking. In addition, certain amino acids, such as tryptophan, may donate electrons to the RB triplet excited state thereby producing amino acid radicals that can further contribute to cross-link formation (Redmond and Kochevar, 2019).

There were little differences in the photobonding properties between the SF-PEG300 and SF-PEG3350 hydrogels. The *ex vivo* bonding strengths were in good agreement with the *in vivo* measurements. SF-PEG3350 hydrogels appear to have slightly higher bonding strengths in some conditions (although differences were not statistically significant), but in the *in vivo* photobonding experiments these appeared to be more breakable under similar forces. For this reason, the *in vivo* proof-of -concept tests were performed with the SF-PEG300 hydrogel.

Excess RB was gradually extracted from the hydrogel over time by the action of tears, leading to increased transparency. In its unstained form, the hydrogel reaches approximately 95% transmittance in the 400–800 nm visible spectrum, as reported in a previous study (Gutierrez-Contreras et al., 2024). The extracted RB did not seem to induce toxicity in the eye, as previously demonstrated even when employing a higher concentration of RB than that used in the present study (Gallego-Muñoz et al., 2017; Lorenzo-Martín et al., 2018).

A comprehensive surgical protocol was developed to enable photobonding of SF hydrogels to corneas. This procedure incorporates optimization of hydrogel dimensions to improve corneal conformity, bonding efficiency, and re-epithelialization; the use of a custom-designed mask to shield the central pupillary region and limbus from irradiation; and refinement of irradiation parameters.

Previous experiments by our group showed that positioning the membrane in close proximity to the limbus resulted in irradiation-induced neovascularization (data not shown). Accordingly, in the present proof-of-concept, the hydrogel was applied approximately 2–3 mm from the limbus to reduce the risk of neo-vessel formation.

The *in vivo* proof of concept confirmed the feasibility of photobonding the hydrogel to a de-epithelialized rabbit cornea and demonstrated progressive re-epithelialization over time.

A key objective of using SF-based hydrogels as substitutes for AM is to promote epithelial closure in severe injuries which, if proper re-epithelialization does not occur, may progress to recurrent ulcers, stromal edema, or fibrosis, ultimately resulting in vision loss. For this reason, it is important to emphasize that in this initial study the hydrogel supported epithelial cell migration and enabled restoration of the epithelial layer. This is an encouraging and noteworthy first achievement for this proof-of-concept study. Moreover, no inflammation or stromal alterations were observed at the evaluated time points. Following this initial study, in which the SF-based hydrogel demonstrated suture-free adhesion and supported re-epithelialization, future studies applying these hydrogels in models of severe corneal damage, such as alkali burns, will allow us to evaluate their true therapeutic potential.

Unlike AM, SF hydrogels lack inherent growth factors. However, growth factors from tears might be absorbed and captured in the hydrogel, enhancing the re-epithelialization of the cornea upon release. The hydrogel could also be soaked with exogenous growth factors, to further mimic the properties of the AM. Ongoing studies investigating the potential of loading SF hydrogels with growth factors show promising results (Fernandez-Gutierrez M, et al. IOVS 2023; 64:ARVO E-Abstract 1883). In addition, our group has found a greater secretion of regenerative and antifibrotic growth factors in an *in vitro* corneal cell culture wound model on SF-PEG300 substrates compared to collagen substrates (Gallego-Muñoz et al., 2025, manuscript submitted).

The presence of nictitating membrane in rabbits posed some challenges, as it occasionally displaced the hydrogel. However, this structure is not present in humans. In fact, the peeling strengths measured in the current study (0.13–0.33 N/cm^2^) are several orders of magnitude higher than the reported maximum time-averaged blink eyelid shear stress (1.6 × 10^−3^ N/cm^2^) (Mutharasan R, et al. IOVS 2002; 43:ARVO E-Abstract 974). Moreover, the photobonding of the hydrogel functions like an adhesive film, exhibiting strong bonding strengths under tension but weaker adhesion when peeling strengths are applied. We expect that this characteristic will allow for easy removal of the hydrogel from the cornea after healing without epithelium damage. Moreover, given the continuous process of epithelial regeneration, spontaneous detachment of the hydrogel may be expected overtime. The loss of the hydrogel during histology processing of the sample in one rabbit in which the epithelial layer had not been removed, may indicate such process. This observation aligns with clinical reports showing, that upon detachment of the AM from the cornea in clinical practice, the epithelial layer typically remains intact (Malhotra and Jain, 2014; Walkden, 2020). Following this initial proof-of-concept that showed successful hydrogel adhesion and re-epithelialization, future studies should include a larger number of animals, assess the long-term behavior of the SF-hydrogel, quantify the clinical follow-up and histopathological findings with an objective scoring system, and expand the application to severe corneal injury models.

This study advances the development of innovative corneal bandages. Enhancing the SF-hydrogels with growth factors could further improve their efficacy, potentially matching the therapeutic benefits of the AM.

## Data Availability

The raw data supporting the conclusions of this article will be made available by the authors, without undue reservation.
